# Povidone-iodine pharmacokinetics and study design

**DOI:** 10.1186/s12886-020-1313-9

**Published:** 2020-01-17

**Authors:** Jagger Koerner, Andrzej Grzybowski

**Affiliations:** 1 Department of Ophthalmology, Wake Forest University, Winston Salem, USA; 2 Department of Ophthalmology, University of Warmia and Mazury, Olsztyn, Poland

**Keywords:** Endophthalmitis prophylaxis, Povidone-iodine, Intravitreal injection

## Abstract

Dr. Gnanasekaran et al. reported the bactericidal activity of various concentrations of povidone iodine (PI) solution in an agar plate experiment of respiratory flora. The study design and the pharmacokinetic properties of PI solution ensured that dilute PI would not be effective in this study. These results may not replicate the typical clinical situation and are significantly different than a previously reported agar plate experiment, again owing to subtle but very significant differences in methodology.

## Main text

We read with interest the study by Gnanasekaran et al., and would like to further discuss its methods and a similar agar plate in vitro study conducted by Silas et al. Gnanasekaran and colleagues exposed blood-agar plates to various concentrations of povidone iodine (PI), decanted excess fluid, waited 30 s and then loaded the plates with respiratory flora [1]. This is very different from loading the plates with bacteria and *then* applying PI solution.

Silas et al. found that by applying PI to blood agar plates *already* loaded with bacteria, 10, 5 and 2.5% PI eradicated all bacteria [2]. Applying 1% PI three times separated by 2 min produced similar results to 5% PI applied once. The differences between these two agar plate studies are the result of the timing of PI application; before or after the bacteria are loaded onto the plate. They also illustrate a contrast with liquid media-based studies, in which dilute PI solution can eradicate high concentrations of virulent bacteria in 15–30 s [3].

The pharmacokinetic properties of PI solution explain these findings; specifically, the reduction of bactericidal free iodine to iodide species after interaction with organic substances. Dilute PI solution has less total available iodine and therefore becomes “spent” or depleted earlier than more concentrated solutions [4].

Bearing this in mind, therefore, it is not surprising that the 1% PI solution did not inhibit bacterial growth at all; its limited free iodine was exhausted well before the lengthy bacterial loading process was complete. The conclusion “ophthalmologists should avoid diluting PI to concentrations lower than 5% for pre-injection antisepsis” is supported by this article only if usual practice consists of applying PI and then contaminating the site for 5 min. In contrast, if the eye is considered “dirty” prior to antisepsis, PI solution applied, and then the site kept clean for 30 s prior to injection, the results of this study are not applicable. In fact, 1% PI solution applied several times prior to injection can produce excellent antisepsis as shown by Silas et al.

Unfortunately, there is no standardized reliable way to simulate the ocular surface to better study this and related topics in vitro. Applying PI solution after bacteria are loaded onto the plates and waiting 30 sec for the solution to work could better simulate the situation in clinic, especially if typical precautions such as not talking and mask wearing are taken.

## Data Availability

Not applicable.

## Abbreviation

PI: Povidone iodine
